# Endovascular stenting of a chronic ruptured type B thoracic aortic dissection, a second chance: a case report

**DOI:** 10.1186/1752-1947-2-41

**Published:** 2008-02-07

**Authors:** Ali Arshad, Sumaira L Khan, Simon C Whitaker, Shane T MacSweeney

**Affiliations:** 1 The Departments of Vascular and Endovascular Surgery, Nottingham University Hospital, Nottingham, UK; 2 The Department of Vascular Radiology, Nottingham University Hospital, Nottingham, UK; 3 The Lodge, Tattershall Drive, Nottingham, NG7 1AX, UK

## Abstract

**Introduction:**

We aim to highlight the need for awareness of late complications of endovascular thoracic aortic stenting and the need for close follow-up of patients treated by this method.

**Case presentation:**

We report the first case in the English literature of an endovascular repair of a previously stented, ruptured chronic Stanford type B thoracic aortic dissection re-presenting with a type III endoleak of the original repair.

**Conclusion:**

Endovascular thoracic stenting is now a widely accepted technique for the treatment of thoracic aortic dissection and its complications. Long term follow up is necessary to ensure that late complications are identified and treated appropriately. In this case of type III endoleak, although technically challenging, endovascular repair was feasible and effective.

## Introduction

Medical therapy has been the mainstay of treatment for uncomplicated Stanford type B aortic dissection for many years [1]. However, more recently, endovascular aortic stenting of dissecting thoracic aneurysm has also become a well recognised treatment option [2]. Ongoing studies are currently investigating the long-term safety and efficacy of this technique. The complications of thoracic aortic stenting are also well recognised and graft perforation following endovascular stenting is a known entity [3,4]. The best treatment modality for the treatment of these complications remains controversial. We report the first case in the English literature of an endovascular repair of a previously stented, ruptured chronic Stanford type B aortic dissection. Our report highlights both the need for awareness of the late complications of endovascular thoracic aortic repair as well as the feasibility of re-stenting in this difficult scenario.

## Case presentation

An 82-year-old man, who had previously undergone the first successful endovascular repair of a ruptured chronic type B dissection, presented to us again five years after his first procedure [5].

His original diagnosis of a Stanford type B aortic dissection had been made in 1994. He was initially managed medically with antihypertensive medication alone, however seven years later, he suffered sudden collapse and chest pain. A ruptured false lumen thoracic aneurysm was diagnosed by spiral computed tomographic angiography (SCTA). The aortic dissection extended distally from the left subclavian artery to the left common iliac artery. The coeliac axis appeared to have a common origin from both the true and false lumens, whereas the left renal and inferior mesenteric arteries originated from the false lumen.

He was deemed to be unsuitable for open surgery due to significant medical co-morbidity, including atrial fibrillation, ischaemic heart disease and chronic obstructive airways disease so therefore endovascular treatment of his condition was undertaken [5]. The challenge was to exclude the rupture while maintaining perfusion of his gut and kidneys. This was undertaken using a total of four Gore Excluder endografts (WL Gore & Associates, Flagstaff, Ariz.). This has been previously described [5].

After a stormy post-operative course, he was discharged home with regular clinical and radiological follow-up, but after two years he declined to attend further review.

**Figure 1:**
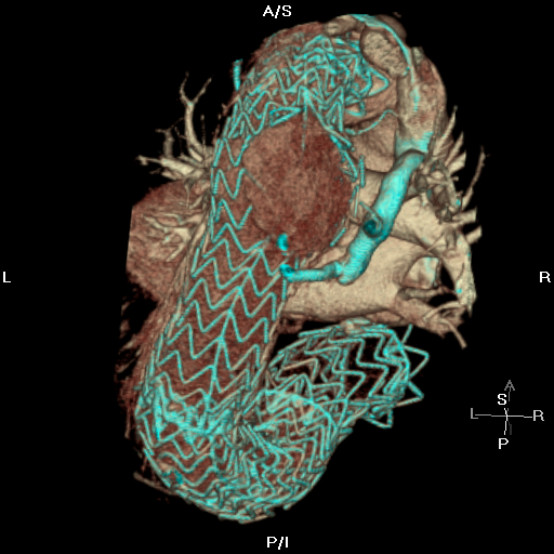
Three dimensional reconstruction showing the type III endoleak from the failed thoracic stent component.

**Figure 2:**
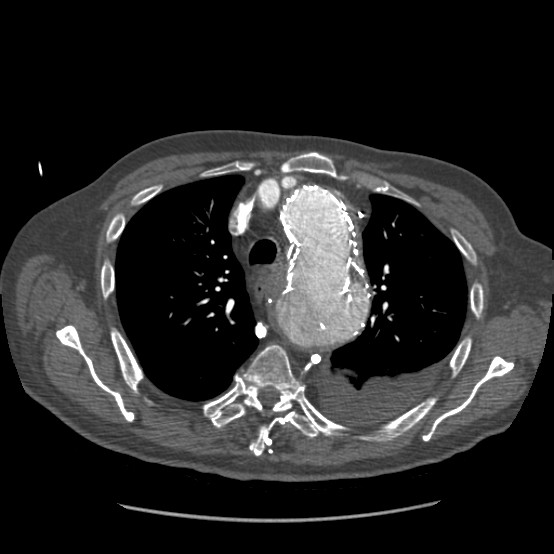
Two dimensional CT image showing posterior defect in the thoracic stent component and associated haematoma in the left thoracic cavity.

Four years after his original procedure, he re-presented to a nearby hospital with a one-month history of increasing chest and back pain associated with shortness of breath. Chest X-Ray showed left lower zone shadowing and he was treated for pneumonia. His respiratory symptoms improved, but his chest and back pain continued. Further laboratory investigations revealed that he was hypercalcaemic with a corrected calcium of 2.77 mmol/l. SCTA revealed a haematoma in the left mid-thoracic cavity associated with vertebral body erosion. The hypercalcaemia was attributed to this bony erosion and it was postulated that this had been caused by the pulsatile haematoma giving rise to his symptoms of chest & back pain. The patient was transferred to the vascular unit at our institution. Three dimensional SCTA reconstruction revealed a probable defect at the junction between the middle two of the four stents (adjacent to the haematoma), which appeared to have only 5 mm of overlap. Above the level of the haematoma, an apparent perforation of the second thoracic stent was seen. The haematoma was thought to be originating from either or both of these structural defects. These defects therefore constituted a type III endoleak [Figure 1, 2]

The initial plan was to perform re-stenting on an urgent basis meanwhile optimising his general medical condition. However, on the third day of admission his condition deteriorated suddenly as he became hypotensive and confused. A decision was made with the patient & his family to perform immediate re-stenting of the thoracic aorta as an emergency.

**Figure 3:**
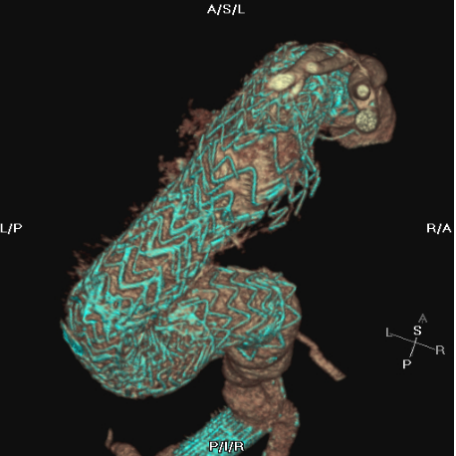
Three dimensional reconstruction following re-stenting showing the new component inside the defective portions of the original repair.

Under general anaesthesia, the right brachial and right common femoral arteries were exposed and cannulated. A standard guidewire was passed from the brachial artery to the right common femoral artery using a snare device from below ensuring that the true lumen was entered and avoiding entering either of the two false lumens in the abdominal aorta. Endovascular repair was then carried out in the standard fashion through the right common femoral artery. A 42 × 200 mm Medtronic Talent endograft (Medtronic, Santa Rosa, Calif.) was deployed across the site of blowout and the presumed defective junction. A further 42 × 150 mm Medtronic Talent endograft was used to overlap and extend distally. SCTA performed the following day demonstrated good stent position with no evidence of endoleak [Figure 3].

The patient's postoperative recovery was complicated by postoperative pneumonia requiring intravenous antibiotics. Despite supportive treatment he eventually succumbed on the ninth postoperative day to this respiratory complication.

## Conclusion

We believe that this case was the first example of endovascular repair of a leaking, previously stented chronic type B ruptured aortic dissection. Similar graft failures have been documented, although these have either been treated medically or surgically by median sternotomy and open repair [6,7].

As endovascular repair of the thoracic aorta becomes more common, it is inevitable that the number of long term complications will increase. This case illustrates the importance of long term follow up even when all appears satisfactory two years post operatively. In this case further follow up had been declined by the patient. An endovascular approach is feasible but can be technically challenging. Careful monitoring of the durability of endovascular repair of ruptured chronic aortic dissection will be needed to determine the role of endovascular repair in this situation.

## Competing interests

The author(s) declare that they have no competing interests.

## Authors' contributions

AA collated the images and co-wrote the manuscript, SK co-wrote the manuscript and conceived the report, SW co-wrote the manuscript and generated the three-dimensional reconstruction images and SM co-wrote the manuscript and was responsible for final approval.

## Consent

Written informed consent was obtained from the patient's next of kin for publication of this case report and any accompanying images. A copy of the written consent is available for review by the Editor-in-Chief of this journal.

## References

[B1] Wheat MJ Current status of medical therapy of acute dissecting aneurysms of the aorta World J Surg 1980 4 563 9 10.1007/BF02401630 7233926

[B2] Eggebrecht H Nienaber CA Neuhauser M Baumgart D Kische S Schmermund A Herold U Rehders TC Jakob HG Erbel R Endovascular stent-graft placement in aortic dissection: a meta-analysis European Heart Journal 2006 27 4 489 498 10.1093/eurheartj/ehi493 16227309

[B3] Piffaretti G Tozzi M Lomazzi C Rivolta N Caronno R Castelli P Complications after endovascular stent-grafting of thoracic aortic disease J Cardiothorac Surg 2006 12 1 26 10.1186/1749-8090-1-26 PMC1574296 16968547

[B4] Cosin O Rousseau H Otal P Cron C Chabbert V Joffre F Late perforation of a thoracic aortic Dacron graft by a metallic stent-graft component J Endovasc Ther 2006 13 5 676 80 10.1583/06-1881.1 17042667

[B5] Hinchliffe RJ Davidson IR MacSweeney STR Endovascular repair of a ruptured chronic type B aortic dissection J Vasc Surg 2002 36 401 3 10.1067/mva.2002.125749 12170224

[B6] Toyama M Usui A Yoshikawa M Ueda Y Thoracic aneurysm rupture due to graft perforation after endovascular stent-grafting via median sternotomy Eur J Cardiothorac Surg 2005 27 1 162 4 10.1016/j.ejcts.2004.09.007 15621493

[B7] Bockler D Schumacher H Ganten M von Tengg-Kobliqk H Schwarzbach M Fink C Kauczor HU Bardernheuer H Allenberg JR Complications after endovascular repair of acute symptomatic and chronic expanding Stanford type B aortic dissections J Thorac Cardiovasc Surg 2006 132 2 361 8 10.1016/j.jtcvs.2006.02.056 16872963

